# Evidence Synthesis via Indirect Treatment Comparisons in the European Framework of Joint Clinical Assessment

**DOI:** 10.3390/epidemiologia7030064

**Published:** 2026-05-05

**Authors:** Alberto de la Cuadra-Grande, María Arruñada, Alejandro García-Solís, Ana Rossignoli-Montero, Miguel Ángel Casado

**Affiliations:** 1 Pharmacoeconomics & Outcomes Research Iberia (PORIB), Paseo Joaquín Rodrigo 4–Letra I, Pozuelo de Alarcón, 28224 Madrid, Spain; adelacuadra@porib.com (A.d.l.C.-G.);; 2 Therapeutic Positioning Report and Health Technology Assessment Area, Medicines for Human Use Department, Spanish Agency of Medicines and Medical Devices (AEMPS), 28022 Madrid, Spain; 3 Fundación PORIB, Paseo Joaquín Rodrigo 4–Letra I, Pozuelo de Alarcón, 28224 Madrid, Spain

**Keywords:** health technology assessment, indirect treatment comparison, joint clinical assessment, matching-adjusted indirect comparison, network meta-analysis, simulated treatment comparison, PICO

## Abstract

The application of the Health Technology Assessment Regulation (HTAR) gives way to joint European work, such as the Joint Clinical Assessment (JCA). This requires the definition of a PICO (Population–Intervention–Comparator–Outcome) question representative of all the member states of the European Union. The key to answering the PICO will be the synthesis of evidence through direct comparisons when there are randomized clinical trials (RCTs) including the same comparators, and via indirect treatment comparisons (ITCs) when comparators differ across RCTs. The aim of this report is to provide a synthesized and clear methodological framework to guide those stakeholders involved in JCAs when interpreting the results of ITCs, including descriptions on: (1) assumptions associated with ITCs; (2) how to select the method for ITC; (3) strengths and limitations associated with the methods; and (4) basics for understanding the method for ITC. This methodological framework could help those health care institutions, patient associations, consumer organizations, health-related nongovernmental organizations, health technology developers, and healthcare professionals involved in JCAs to better understand ITCs and incorporate this evidence into decision-making.

## 1. Introduction

Health technology assessment (HTA), as we currently know it, has undergone a paradigm shift with the phased implementation of the Health Technology Assessment Regulation (HTAR). On 12 January 2025, this regulation began to be applied to the assessment of new cancer drugs and advanced therapies. From 2028 to 2030, it will be applied to orphan drugs and to all other drugs, respectively [1].

The aim of the HTAR is to ensure the efficient use of resources and avoid duplication of efforts by centralizing clinical assessment and making it unique for the entire European Union, which means that joint clinical assessments (JCAs) will be conducted. Although clinical assessment is unique for the entire European Union, Member States remain solely responsible for national HTA processes, conclusions on the value of health technologies, and decisions derived from these HTAs [1].

In this context, the implementation of HTAR requires the creation of a governance structure, highlighting the Health Technology Assessment Coordination Group (HTACG), which is composed of representatives from the HTA authorities and bodies of each Member State. This group must ensure that the collaborative scientific work, procedures, and methodologies for the preparation of JCA reports offer maximum quality guarantees, are prepared in a timely manner, and reflect an evolved state of the art in medical science [1].

This governance structure relies on the HTACG, which will be supported by the European Commission (acting as Secretariat) and the subgroups with which it works closely. In addition, the stakeholder network, consisting of patient associations, consumer organizations, health-related nongovernmental organizations, health technology developers (HTDs), and healthcare professionals, will be able to support the work of the HTACG and its subgroups [1].

The implementation of JCAs is not exempt from challenges, such as establishing PICO (Population–Intervention–Comparator–Outcome) questions that are appropriate for the needs of different Member States [2]. Given that healthcare systems and clinical practices vary between Member States, it is difficult to use the same comparator for the PICO question in all countries, among other concerns. In this context, considering that evidence-based information can be obtained from different studies, evidence synthesis is key in terms of JCA [3,4].

Several guidelines adopted by the HTACG have been published [3,4], which seek to provide methodological guidance to those evaluators responsible for preparing the JCA report and to guide HTDs in identifying limitations and addressing biases and uncertainties in direct and indirect treatment comparisons (ITCs). These comparisons are key to enabling PICO questions to be answered and, therefore, ensuring that JCAs are conducted based on data on the relative efficacy and safety of health technologies derived from different studies [3,4].

Thus, the aim of this report is to synthesize the methodology guidelines published by the HTACG for conducting ITCs [3,4], and to elaborate on the proposed methods to provide all stakeholders who will be involved in JCAs with a structured methodological framework that serves as a basis for the use and interpretation of the results of ITCs.

## 2. Methods

This report was based on a thorough review and synthesis of the guidelines published by the HTACG for conducting ITCs [3,4]. Additionally, to elaborate on the various proposed methods, a narrative review of the scientific literature was conducted, which included other guidelines and articles describing or analyzing methods for ITCs.

A working group composed of experts in biostatistics, health economics and outcomes research (HEOR) and HTA was established to develop a precise methodological framework, critically analyze the strengths and limitations of ITC methods, and provide those stakeholders involved in JCAs with a rigorous and understandable description.

## 3. Synthesis of Scientific Evidence

In HTA, and within the framework of JCA, randomized clinical trials (RCTs) represent the highest level of scientific evidence [3,4]. However, RCTs represent the only evidence available in many cases, mainly due to the innovative nature of the technologies being evaluated. When the findings of various RCTs are combined, they may all include the same comparators. In these cases, the synthesis of evidence consists of a direct comparison via meta-analysis techniques [3,4].

In contrast, RCTs may compare different interventions with each other. For example, there may be one RCT comparing alternative “A” vs. “B” and another comparing “A” vs. “C”, revealing a lack of evidence comparing “B” vs. “C” in a head-to-head study. Within this context, ITCs are used to compare different interventions via data from independent studies [3,4].

ITCs are called “adjusted” when the results of the treatments included in the different RCTs are adjusted based on a common comparator. Their main strength is that they preserve the random allocation of study interventions, maintaining a relatively homogeneous distribution of known and unknown confounding factors. Methods that compare individual treatment arms from different RCTs or that include non-randomized studies (observational or single-arm interventional studies) are considered “naïve” ITCs and should be avoided due to their high risk of bias, particularly confounding and selection bias arising from the loss of randomization [3,4].

When conducting an ITC, it is essential to verify the assumption of exchangeability, whereby if the individuals in one RCT are replaced by those in another RCT, the observed treatment effect would be the same. This assumption is based on the properties of similarity and homogeneity [3,4].

Similarity refers to an equivalent distribution of effect-modifying factors between RCTs. To evaluate this property, a critical and comprehensive review of the studies must be performed, including their design, the clinical and sociodemographic characteristics of the participants, interventions, and comparators or definitions of outcome variables, among others [3,4].

Homogeneity refers to the relative efficacy of interventions between treatment–comparator pairs being constant across RCTs. Although there are statistical tests to evaluate this property (Cochran’s Q or I^2^), providing their results with a critical review of the methodology used is essential [3,4].

Finally, consistency represents an additional property to be evaluated, which refers to the agreement between results obtained from direct and indirect comparisons. Comparing direct effects with indirect estimates is the simplest technique for studying consistency, although alternative techniques can be used [3,4].

## 4. Selection of the Method for Indirect Treatment Comparison

**Figure 1:**
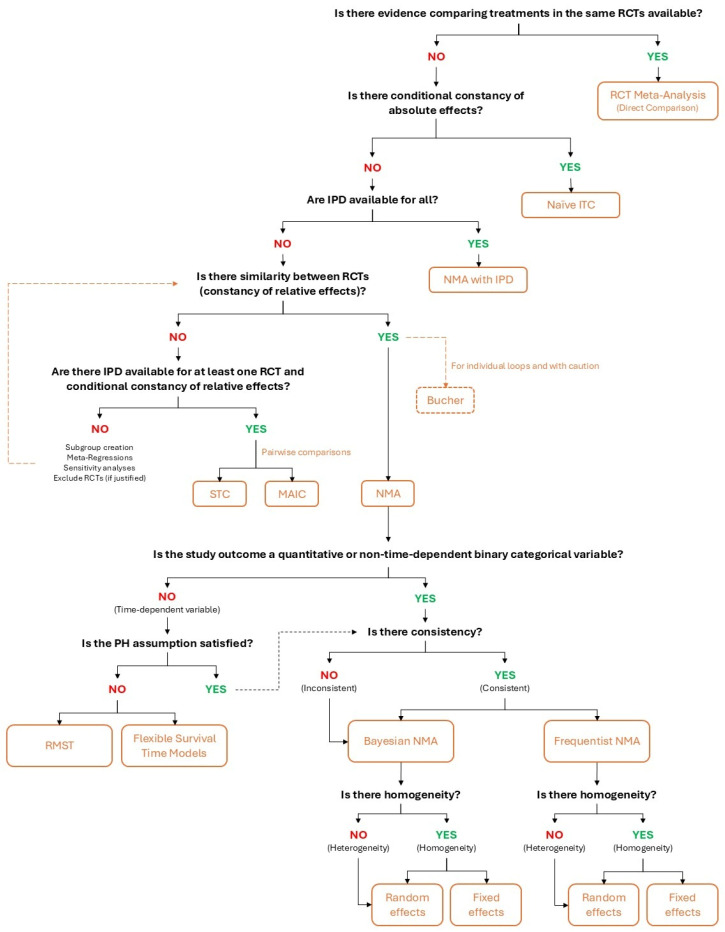
Flowchart for selecting the ITC methods. HTACG: Health Technology Assessment Coordination Group; IPD: individual participant data; ITC: indirect treatment comparison; MAIC: matching-adjusted indirect comparison; NMA: network meta-analysis; PH: proportional hazards; RCT: randomized clinical trial; RMST: restricted mean survival time; STC: simulated treatment comparison. Figure created by the authors on the basis of the guidelines published by HTACG [3,4].

There are different types of ITCs [5,6,7,8,9,10]; thus, selecting an appropriate method is essential (Figure 1). Furthermore, in HTA, it is common to use direct comparisons, aggregating all evidence from direct comparisons via meta-analysis, and indirect comparisons are made from these aggregated data [3,4].

The key drivers for selecting the most appropriate ITC methodology include the availability of individual participant data (IPD) and the similarity between RCTs, among others [3,4]. Whenever IPDs are available for all RCTs considered, network meta-analysis (NMA) with IPD could be used [5]. However, the complexity of collecting all IPDs implies that this technique is suitable only in very specific contexts [5].

In contrast, the most common situation in HTA is to rely on aggregated data from RCTs. In these circumstances, the Bucher method [6] can be conducted after a rigorous determination of the similarity between RCTs [3,4]. Nonetheless, it is common to use more sophisticated techniques, such as NMA with aggregated data from RCTs [7]. Population-adjusted methods, such as matching-adjusted indirect comparison (MAIC) [8,9] or simulated treatment comparison (STC) [10], represent alternative methods, although they should be applied with caution [3,4]. The choice between NMAs with pooled data [7] or population-adjusted methods [8,9,10] depends on the similarity between RCTs [3,4].

When similarity between studies is demonstrated, the constancy of relative effects can be assumed, which considers that all effect modifiers are balanced across trials [3,4], and NMA techniques can be used [7]. Traditional frequentist NMA models allow the estimation of a weighted average effect and its confidence interval. However, the development of increasingly powerful statistical software has encouraged the use of Bayesian NMAs that model heterogeneity by using probability distributions [7]. Both models are applicable to all types of variables, except for time-dependent events not satisfying the proportional hazards (PH) assumption, in which case it is advisable to use restricted mean survival time (RMST) or flexible survival time model methods [3,4].

In those cases where substantial differences between RCTs are observed that call into question their similarity, the conditional constancy of relative effects should be demonstrated, which assumes that relative treatment effects are constant across RCTs after adjusting for those effect modifiers during the analysis [3,4]. In these cases, the MAIC [8,9] and STC [10] could be the methods of choice if IPDs are available from at least one of the studies [3,4], as these methods aim to homogenize the characteristics of the participants in both RCTs, thus allowing effect-modifying factors to be redistributed equally, which, in an artificial manner, increases similarity [3,4].

The conditional constancy of absolute effects is a stricter assumption that can be considered when conducting a population-adjusted method, which assumes that absolute treatment effects are constant across studies when imbalanced effect modifiers are adjusted [3,4]. Although this assumption can be replaced by the conditional constancy of relative effects when conducting population-adjusted ITCs, which is more relaxed, it should be required in those analyses involving naïve ITCs [3,4].

Finally, in those cases where none of the methods described can be applied, other approaches could be considered (i.e., estimating results by patient subgroups, excluding specific RCTs with proper justification, performing sensitivity analyses, or employing meta-regression techniques) [3,4].

In addition to the assumptions that should be satisfied when conducting the different methods for ITCs, other aspects should also be considered when selecting the most appropriate approach, including their strengths and limitations or the type of variable used for measuring treatment effects (Table 1).

## 5. Description of Methods for Indirect Treatment Comparison

### 5.1. Bucher Method

**Figure 2:**
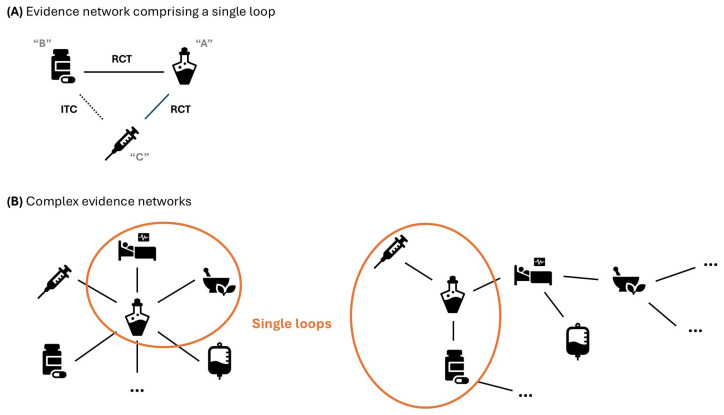
Examples of evidence networks. ITC: indirect treatment comparison; RCT: randomized clinical trial. The letters “A”, ”B” and ”C” are the names of the hypothetical comparators.

The Bucher method allows estimation of the effect of treatment “B” vs. “C” with “A” as the common comparator. Although this method is usually recommended for single loops including three treatments, with one of them being the common comparator (Figure 2A), it can be used in more complex evidence networks by their decomposition into single loops (Figure 2B).

The indirect effect is estimated as the sum of the direct effects for each pair of interventions on a logarithmic scale. The measure of association is estimated as the sum of the χ^2^ (chi-squared) tests, whose degrees of freedom correspond to the number of studies available for each direct comparison [6].

Even though estimates from the Bucher method could be considered relatively robust, certain factors may influence the results [3,4,6]:

- Differences in weights provided to each study in those direct vs. indirect comparisons can “artificially duplicate” the sample size in some RCTs.
- Differences in the design of the studies compared might yield different treatment effects.
- Differences in the method for measuring the result could produce differences in the observed treatment effect.
- Differences in the distribution of effect modifiers could impact the outcomes (e.g., overrepresentation of certain subgroups in one study compared to another), which could limit the consistency of the relative effects assumption.

### 5.2. Network Meta-Analysis (NMA)

Most ITCs, including NMAs, can be performed in a frequentist or Bayesian framework. While frequentist approaches provide deterministic estimates along with their confidence intervals (CI) based on the likelihood or probability of the study data to be observed in the study population, Bayesian analyses rely on probability distributions of the observed data with a conditional probability derived from previous knowledge, which is described by credible intervals (CrI) [11,12]. Thus, a 95% CI in a frequentist framework indicates that if the experiment is repeated 100 times, 95% of the CIs estimated would include the true estimate, which remains unknown [12]. In contrast, a 95 CrI suggests that, based on the observed data, there is a probability of 95% to find the true effect value within the interval estimated [12].

In addition, both fixed and random effects models can be designed for ITCs [13,14,15,16,17]. Fixed-effects models assume that there is a true (unknown) effect, the study populations in each RCT being a sample of this hypothetical common population. Under this assumption, the differences in effect sizes between RCTs are produced by random errors at sampling, which can be quantified by the intra-study variance (S_intra_^2^). This implies that, if the sample of the RCTs tends to be infinite (N~∞), reflecting the whole universe of study, all RCTs would estimate the same treatment effect. For this reason, the fixed-effects model relies on the inverse of intra-study variance (S_intra_^2^) to calculate the aggregate effect, which means that studies including greater sample sizes (↑ N) are associated with less intra-study variance (↓ S_intra_^2^), thus having a greater impact on the estimate (↑ weight in the ITC) [13,14,15,16,17].

In contrast, random-effects models consider that the true (unknown) effect could vary across studies, not only due to random errors caused by sampling (S_intra_^2^), but also because systematic differences could be found between populations (e.g., participants’ age, socioeconomic status, clinical profile…), yielding an inter-study variance (S_inter_^2^). The contribution of each study in a random-effects model depends on both intra- and inter-study variances, potentially providing more weight to those studies with smaller sample sizes and wider uncertainty intervals (CI or CrI) [13,14,15,16,17]. Given these assumptions, random-effects models could represent a more appropriate alternative when remarkable heterogeneity between studies is found [3,4].

#### 5.2.1. Frequentist Network Meta-Analysis (NMA)

In the case of frequentist NMAs, all direct comparisons between the same pair of interventions are first aggregated via different methods, depending on whether a fixed- (e.g., inverse variance, Mantel–Haenszel, Peto, β-binomial, etc.) [14,18] or random-effects (e.g., DerSimonian–Laird [DSL], Knapp–Hartung [KH], etc.) [19,20] model was selected. Subsequently, indirect comparisons are conducted with an approach similar to the Bucher method [14], thus requiring individual loops, star networks (a comparator common to all interventions), or ladder application [3,4]. Otherwise, there are more complex methods for designing frequentist models [3,4].

#### 5.2.2. Bayesian Network Meta-Analysis (NMA)

Bayesian NMAs, including fixed- and random-effects models, are mostly programmed in WinBUGS or OpenBUGS, both derived from BUGS (Bayesian inference using Gibbs sampling), a software commonly used to develop models called MCMC (Markov Chain–Monte Carlo). However, there are other tools for designing Bayesian models currently available (e.g., R, STATA, etc.) [15,21,22,23].

Markov models (Markov chains) simulate processes using mutually exclusive Markov nodes or states that communicate with each other using probabilities. In addition, Monte Carlo simulation consists of repeating the same simulation based on probability distributions numerous times, which informs on the probabilistic trend of the results [21,22,23]. For instance, when flipping a coin to see if it lands on heads or tails, if it is flipped two times, it can land once on heads and once on tails, but two heads or two tails can be frequently observed. When the coin is flipped 10 times, the distribution between heads and tails will be closer to 50:50, and even more so if the coin is flipped 1000 or 1,000,000 times. This trend can be captured by conducting Monte Carlo simulations.

Bayesian NMA models are fed with probability distributions for each parameter, which are generally noninformative to allow a wide range of values for the target variable [22,23]. The models are adjusted based on the effects observed in the RCTs, and the most appropriate model, which will provide the NMA results, should be selected based on statistical criteria, including Akaike (AIC), Bayesian (BIC), and Deviation (DIC) information criteria, among others [21,22,23].

#### 5.2.3. Network Meta-Analysis (NMA) of Time-to-Event Variables

Those treatment effects measured as time-to-event can be considered in NMAs when data satisfy the proportional hazard (PH) assumption [24]. This means that the hazard of the event is proportional over time; thus, the hazard ratio (HR) could be considered relatively constant [24]. The first constraint associated with NMA of time-to-event variables is that IPD are needed to assess the PH assumption. Given the difficulties of accessing all IPD, a method has been proposed to allow for the “reconstruction” of IPDs by using the published Kaplan–Meier curve [25].

Whenever the PH assumption is unmet, RMST [26] or flexible survival time models [27,28,29,30] have been proposed to allow the inclusion of these data in NMAs.

The RMST is defined as [26]

(1) $$\mu =E(x)=E[\min(T,\;t*)]=\int_{0}^{t*}S(t)\mathrm{dt},$$

which means that time X = min(T, t*), where “T” is the outcome, limited to a time horizon t* > 0, which equals the area under the curve (AUC) from t = 0 to t*. In this way, a t* can be selected in the identified RCTs where the PH assumption is met and, consequently, NMAs can be conducted considering these time horizons (even if the assumption is not met for the complete horizons) [26].

Flexible survival models are nonparametric models (e.g., fractional polynomials, piecewise exponential [PWE] models, etc.), which are alternatives to conventional models (Cox, exponential, Weibull, etc.) for studying HR in time-dependent events [27,28,29,30].

The polynomial proposed for modeling time-dependent events is [27,28]

(2) $$y=\beta o+\beta 1t^{p1}+\beta 2t^{p2}log\left(t\right)$$

Its parameters are adjusted as closely as possible to the RCT data. Subsequently, the estimates provided by these models can be included in the NMA [27,28]. In this approach, choosing the right model is critical, which can rely on statistical criteria (AIC, BIC, DIC, …), best visual fit, and/or clinical plausibility. In any case, the results of sensitivity analyses using alternative polynomials should always be included [3,4].

PWE models divide study time into fragments such that each fragment satisfies the PH assumption (even if this is not satisfied over the entire time horizon). The results of each fragment can be included in an NMA [29,30]. In this case, the number of fragments into which the time horizon is divided can be controversial. Thus, it should always be prespecified and justified in the study protocol, and preferably, published methods should be followed to establish cutoff points. It is advisable to consult with clinical experts, and results should always be accompanied by sensitivity analyses using alternative cutoff points [3,4].

There are other flexible models [31,32,33], although the two described above are the ones mainly recommended in the JCA framework [3,4].

#### 5.2.4. Network Meta-Analysis (NMA) with Individual Participant Data

NMAs with IPDs represent a robust methodology for synthesizing direct and indirect evidence. They do not consist of grouping all IPDs as if they were a single macro study; rather, it is essential to preserve the IPD clusters belonging to each RCT [5]:

- Two-step method: The IPDs for each RCT are analyzed, and the NMA is performed using the estimated pooled data.
- One-step method: IPDs are modeled considering the RCT to which they belong as an additional variable in the model.

Both methods generate relatively similar results, although the “one-step” method could be more convenient when designing a single model, as it complicates the interpretability of the results [5].

### 5.3. Population-Adjusted Indirect Comparisons

Some key concepts arise from population-adjusted indirect comparisons that should be addressed. First, the population resulting from the adjustment may comprise a smaller sample when compared to the original RCT. Given that the decreased number of participants might impair the power for detecting true differences [3,4], the effective sample size (ESS) should always be reported, which refers to the sample size available for conducting the ITC [34].

The concept of ESS brings up another term, which should be explained for a better understanding of population-adjusted methods. When conducting population-adjusted methods, the population included in the RCT-IPD should be as similar to those participants in the RCT-aggregate as possible, which is called overlap, and preferably, the RCT-IPD should be less restrictive than the RCT-aggregate. These requirements are due to the exclusion of all data concerning participants who could not have been in the RCT-aggregate. For example, if the RCT-aggregate only includes participants aged over 40 years and the RCT-IPD enrolled people aged over 65 years, it will be impossible to adjust the populations aged between 40 and 65, which might represent a treatment effect modifier [34]. While STC is less strict with overlap requirements [10,34], MAIC is a very sensitive method in this regard [8,9,34].

Finally, the difference between effect modifiers and prognostic variables should be highlighted. Effect modifiers refer to those covariates that alter the relative effect of the study treatment versus its comparator [35]. In contrast, prognostic variables affect absolute outcomes of both the study treatment and comparator [35]. The methods used to identify effect modifiers and prognostic variables include expert panels, statistical analyses, or previous evidence published in scientific literature [36,37,38,39], which should be clearly described, as it could represent a key aspect of the ITC [3,4].

#### 5.3.1. Matching-Adjusted Indirect Comparison (MAIC)

**Figure 3:**
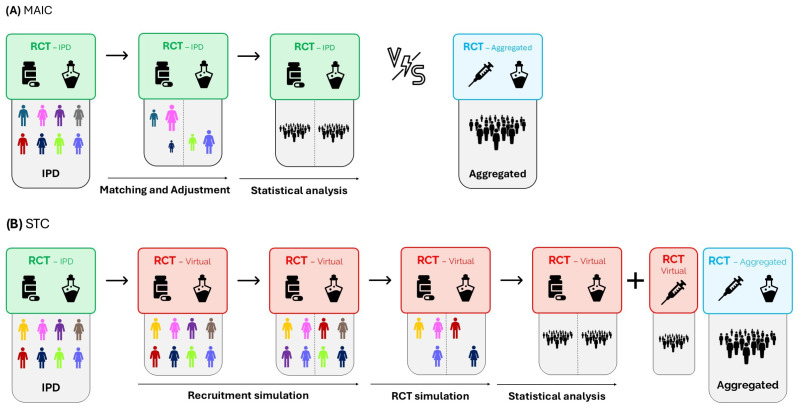
Graphical representation of population-adjusted indirect comparisons. IPD: individual participant data; MAIC: matching-adjusted indirect comparison; RCT: randomized clinical trial; STC: simulated treatment comparison.

The MAIC method first selects those participants from the RCT-IPD who could have been recruited into the RCT-aggregate according to its inclusion and exclusion criteria (matching). Next, using propensity score techniques, individuals in the RCT-IPD are weighted according to their probability of appearing in the RCT-aggregate based on baseline characteristics that are considered relevant effect modifiers (adjusted). The results of the selected and adjusted population of the RCT-IPD arms can be estimated using conventional statistical analyses. These new estimates can be compared by using ITC techniques, such as the Bucher method or NMAs (Figure 3A) [8,9].

#### 5.3.2. Simulated Treatment Comparison (STC)

The STC method performs a discrete event simulation, usually probabilistic (Monte Carlo), of those participants included in the RCT-IPD based on the covariates (effect modifiers) observed in the RCT-aggregate. Clinical outcomes are estimated via a regression model (linear for quantitative variables, logistic for non-time-dependent binary nominal variables, Cox for time-dependent variables, etc.). The models always include the coefficient associated with the patient being included in the RCT-IPD or the RCT-aggregate. The result of the STC is a new “virtual” arm that can be analyzed as if it were a comparator arm of the RCT itself (Figure 3B) [10].

## 6. Critical Assessment of Indirect Treatment Comparisons

A series of practical considerations that may be helpful when interpreting the results of an indirect comparison is presented in Table 2. In this regard, previous guidelines have been published and provide some additional key aspects to critically analyze ITCs that could be helpful for a better understanding of these methods [3,4,16,40]. The present report provides complementary information to those guidelines [3,4,16,40], as it includes conceptual descriptions, which were supposed to be “friendly” in terms of minimizing technical jargon that hinders clear understanding of the methods.

## 7. Conclusions

In summary, evidence synthesis and ITC are key tools in decision-making. However, multiple methodologies and numerous factors must be considered during their interpretation and implementation. For this reason, the European JCA framework offers an alternative for systematic and standardized evaluation of all available evidence on a therapeutic innovation. In addition, it would allow the HTDs to be asked for the protocol of their RCTs, as well as their results in more detail. This is an added advantage for the critical evaluation of the available evidence compared with other contexts in which the synthesis of evidence is based exclusively on data published in scientific articles.

## Figures and Tables

**Table 1:** Overview of methods for ITC.

Method	Requirements	Effect Variable	Strengths	Limitations
Bucher method [6]	Rigorous assessment of similarity between RCTs (consistency of relative effects) An evidence network that comprises a single loop	Categorical (binary: OR, ratio, risk difference, standardized differences) Time-dependent events (HR)	Relatively “simple” and interpretable method Robust results when confounding factors are controlled due to randomization (adjusted ITC)	Exclusive method for evidence networks consisting of individual loops High sensitivity to methodological differences in design, description of the outcome, or subgroup variations between RCTs
Frequentist NMA [7]	Similarity between RCTs (consistency of relative effects) Consistent evidence network	Quantitative (discrete and continuous) Categorical (binary) Time-dependent events (HR) ^A^	Relatively “simple” method (fixed effects models are easier to understand compared to random effects models) There are libraries for statistical software that facilitate the design of these frequentist NMA models (e.g., the “netmeta” library in R) They provide a deterministic result and its CI, which is generally easier to interpret	Requirement of individual loops, star networks, or the application of indirect ladder comparisons They do not reflect the uncertainty associated with heterogeneity The requirement of the consistency assumption must be satisfied
Bayesian NMA [7]	Similarity between RCTs (consistency of relative effects)	Quantitative (discrete and continuous) Categorical (binary) Time-dependent events (HR) ^A^	They reflect the uncertainty inherent in heterogeneity among RCTs It is considered that they provide more robust results compared with frequentist NMAs They have more relaxed application requirements (they do not need to verify consistency to be used) They provide additional methods for studying consistency (e.g., node-splitting) They enable the creation of rankings of the interventions considered	They require advanced knowledge of statistics and programming for their development Its interpretation is less intuitive than in the case of frequentist NMAs The results are accompanied by CrI, a measure with which evaluators are less familiar
RMST [3,4]	NMA requirements and time-dependent outcome	Time-dependent events (when the PH assumption is not satisfied)	Offers the possibility of performing NMA with time-dependent data when the PH assumption is not satisfied	There is considerable uncertainty regarding the selected t*, which may depend on the data available in the RCTs; the t* should always be prespecified in the study protocol and should always be accompanied by sensitivity analyses varying the t* They require advanced knowledge of statistics and programming for their development Its interpretation is not very intuitive, as the time horizon is divided into “sections”
Flexible survival time models [3,4]	NMA requirements and time-dependent outcome	Time-dependent events (when the PH assumption is not satisfied)	Offers the possibility of performing NMA with time-dependent data when the PH assumption is not satisfied	To calculate this, it is necessary to access the IPDs (or reconstruct them from the published Kaplan–Meier curves) ^B^ They require advanced knowledge of statistics and programming for their development Their interpretation is not very intuitive Polynomial models use results predicted by models adjusted using the results of RCTs, rather than the results of RCTs directly PWE models provide fragmented data, and their subsequent aggregation across the entire time horizon is conceptually complex In polynomial models, choosing the right model is critical In PWE models, the number of fragments into which the time horizon is divided can be controversial
NMA with IPD [5]	Access to IPD from all RCTs	Quantitative (discrete and continuous) Categorical (binary) Time-dependent events (HR) ^A^	The definition of consistent inclusion and exclusion criteria between RCTs is facilitated Missing data might be considered in the analysis The results of the individual RCTs can be verified during the analysis Updated follow-up information (in some cases beyond published data) might be included Duplicated participants between the data from each RCT can be identified Statistical analyses can be standardized between RCTs Checking assumptions can be conducted in an easier manner Baseline characteristics can be homogenized (including effect-modifier factors) Results can be estimated for subgroups of interest	Laborious method that requires significant resources (time, personnel, costs, etc.) It may be difficult to contact all authors of published and unpublished RCTs (and obtain access to IPDs) They require advanced knowledge of statistics They pose an ethical component to consider, since IPD is used instead of aggregated data (this type of NMA must be authorized by an Ethics Committee) The results are subject to bias if relevant RCTs are removed due to a lack of access to IPDs The quality of IPDs is not always adequate
MAIC [8,9]	Absence of similarity between RCTs but conditional constancy of relative/absolute effects Availability of IPD from at least one RCT Verification of the assumption of consistency of relative effects Presence of overlap between populations to be compared (populations that are as similar as possible) It is advisable for RCTs with IPD to have a large sample size.	Quantitative (discrete and continuous) Categorical (binary) Time-dependent events (HR)	More robust results are provided by this method when substantial differences in the characteristics of the RCT populations are observed This method is accepted in the scientific community, specifically in HTA, because it has been widely used	High overlap is required because it reduces the sample size and thus the statistical power The inclusion and exclusion criteria for the RCT-IPD should be less restrictive than the criteria for the RCT-aggregate The correct identification of the effect-modifier factors for which the populations will be adjusted is required (it is advisable to seek the collaboration of expert clinicians) The adjusted population may not be representative of the population on which the HTA decision is made
STC [10]	Absence of similarity between RCTs but conditional constancy of relative/absolute effects Availability of IPD from at least one RCT Verification of the assumption of consistency of relative effects Preferably in cases where the RCT with IPD has a small sample size	Quantitative (discrete and continuous) Categorical (binary) Time-dependent events (HR)	More robust results are provided by this method when substantial differences in the characteristics of the RCT populations are observed Aspects related to the design and implementation of RCTs can be simulated and adjusted (e.g., recruitment process) The sample size of the RCT-IPD is maintained after the population adjustment, being the preferred method when there are few patients in the RCT	The method validity depends on the correct specification of the outcome in the regression model The identification and inclusion of all effect-modifier factors are required to be included in regression models The adjusted population may not be representative of the population on which the HTA decision is made

CI: confidence interval; CrI: credible intervals; HR: hazard ratio; HTA: health technology assessment; IPD: individual participant data; ITC: indirect treatment comparison; MAIC: matching-adjusted indirect comparison; NMA: network meta-analysis; OR: odds ratio; PH: proportional hazards; PWE: piecewise exponential; RCT: randomized clinical trial; RMST: restricted mean survival time; STC: simulated treatment comparison. ^A^ Exclusively for those time-dependent variables where the PH assumption is satisfied or the time-dependent variable data have been adjusted via techniques such as RMST or flexible survival time models. ^B^ The Guyot method allows for the accurate “reconstruction” of IPDs.

**Table 2:** Practical considerations for a critical assessment of ITCs.

Practical Considerations			Critical Assessment
1	General considerations and rationale for the need for an ITC		Are the studies included in the evidence synthesis an adequate reflection of the PICO? How was the evidence network generated? Was any comparator discarded? In this case, was its exclusion justified based on the PICO?
2	Assumptions	Similarity	How were potential effect modifiers identified by the authors of the ITC? Is this method suitable for identifying all potential effect modifiers? Are effect modifiers well characterized (i.e., direction and magnitude of interaction)? Do the reported variables include all potential effect modifiers? Did the authors discuss the potential effect modifiers missing? What is the statement of the authors in relation to the assumption of similarity? Did they discuss their findings in this regard? Are the authors’ conclusions regarding similarity aligned with the reader’s critical assessment?
		Homogeneity	Was homogeneity for all pairwise comparisons assessed (and these results are presented)? How was homogeneity assessed? Was the model selection discussed and justified based on homogeneity? Did the authors discuss the need for sensitivity analyses based on their findings in relation to homogeneity? Are the authors’ conclusions regarding homogeneity aligned with the reader’s critical assessment?
		Consistency	Was consistency assessed by the authors? How was consistency evaluated? Was the appropriateness of the methods for assessing consistency discussed? How did the authors define the criteria for violations of consistency? Did the authors discuss the extent of the inconsistency and potential uncertainty in this regard? Are the authors’ conclusions regarding consistency aligned with the reader’s critical assessment?
3	Missing data		Did the authors provide information on the amount of missing data? How did authors deal with missing data? Did they discuss the potential impact of missing data on their results?
4	Direct comparison		Did the authors discuss the appropriateness of conducting a meta-analysis with all direct evidence available? Is the method for direct comparison appropriate (i.e., frequentist vs. Bayesian framework, fixed- vs. random-effects model, etc.? In the case of Bayesian methods, how were prior probability distributions selected? Did the authors justify their approach? Did the authors conduct sensitivity analyses?
5	ITC	General aspects	Is the evidence network well characterized (i.e., number of studies per comparison)? Did the authors discuss the method selection and provide a rationale for their decision? Do results include all relative effect estimates for each comparison, including uncertainties (CI or CrI) and *p*-values? Are results estimated via direct vs. indirect methods available when possible (i.e., assessment of consistency)? If possible, did the authors include rankograms (estimated order of the comparator for being the most effective intervention, based on SUCRA, cumulative probability curves or p-score, among others)?
		NMA	In the case of Bayesian NMAs, is the convergence of Markov chains assessed (demonstration of how the results stabilize around the probabilistic trend as the number of Monte Carlo simulations performed increases)? In time-to-event variables: Did the authors assess the PH assumption? How was the PH assumption? Was the authors’ decision discussed and justified? For flexible parametric models, did the authors discuss and justify the model selection? Was the model selection appropriate based on fit metrics or other information? Were the base-case results compared with those estimated by other models (e.g., visual representation or fit metrics, among other)? For RMST, did the authors discuss and justify the follow-up time selection? Were sensitivity analyses prespecified and justified? Were the estimated HRs compared with those observed?
		MAIC	Was the need for a population-adjusted method discussed and justified? How were relevant effect modifiers identified and selected? Does the author’s selection of effect modifiers align with the reader’s critical assessment? Were the population’s characteristics compared before and after the adjustment? Is the ESS provided, and potential limitations in overlap and statistical power were discussed?
		STC	Was the need for a population-adjusted method discussed and justified? How were relevant effect modifiers identified and selected? Does the author’s selection of effect modifiers align with the reader’s critical assessment? Was the regression model developed for the outcomes estimate described and justified?

CI: confidence interval; CrI: credible interval; ESS: effective sample size; HR: hazard ratio; ITC: indirect treatment comparison; MAIC: matching-adjusted indirect comparison; NMA: network meta-analysis; PH: proportional hazards; PICO: Population–Intervention–Treatment–Outcome; RMST: restricted mean survival time; STC: simulated treatment comparison; SUCRA: surface under the cumulative ranking curve.

## Data Availability

No new data were created or analyzed in this study. Data sharing is not applicable to this article.

## Abbreviations

The following abbreviations are used in this manuscript:

AIC	Akaike information criterion
AUC	Area under the curve
BIC	Bayesian information criterion
CI	Confidence interval
CrI	Credibility interval
DIC	Deviation information criterion
DSL	DerSimonian–Laird
HR	Hazard ratio
HTA	Health technology assessment
HTACG	Health technology assessment coordination group
HTAR	Health technology assessment regulation
HTD	Health technology developer
IPD	Individual participant data
ITC	Indirect treatment comparison
JCA	Joint clinical assessment
KH	Knapp–Hartung
MAIC	Matching–adjusted indirect comparison
NMA	Network meta-analysis
OR	Odds ratio
PH	Proportional hazards
PICO	Population–Intervention–Comparator–Outcome
PWE	Piecewise exponential
RCT	Randomized controlled trial
RMST	Restricted mean survival time
STC	Simulated treatment comparison
SUCRA	Surface under the cumulative ranking curve
